# Preoperative Blood Lactate Level as a Simple Point-of-Care Predictor of Surgical Mortality in Acute Type A Aortic Dissection

**DOI:** 10.5761/atcs.oa.25-00087

**Published:** 2025-08-30

**Authors:** Hirohiko Akutsu, Koji Kawahito

**Affiliations:** Department of Cardiovascular Surgery, Jichi Medical University School of Medicine, Shimotsuke, Tochigi, Japan

**Keywords:** acute type A aortic dissection, lactate, aorta

## Abstract

**Purpose:**

Rapid risk stratification is crucial in patients with acute type A aortic dissection (ATAAD), particularly those presenting with circulatory collapse or malperfusion. This study investigated whether preoperative blood lactate levels could predict surgical outcomes.

**Methods:**

A retrospective analysis was conducted on 166 patients who underwent emergency surgery for ATAAD between 2014 and 2022. Preoperative arterial lactate levels were measured at admission. Multivariate logistic regression identified risk factors for in-hospital mortality. The optimal lactate cutoff value was determined using receiver-operating characteristic curve analysis. Correlation with the Penn classification was also assessed.

**Results:**

In-hospital mortality was 4.2%. A lactate level ≥3.7 mmol/L was independently associated with in-hospital mortality (hazard ratio, 1.41, p = 0.026) and was strongly correlated with Penn classes Ac and Abc. Patients with elevated lactate levels had more severe clinical presentations, prolonged intensive care unit stays, and more postoperative complications. Long-term mortality was also significantly higher in the high-lactate group (p = 0.013).

**Conclusions:**

A preoperative lactate level ≥3.7 mmol/L is a practical and effective point-of-care predictor of surgical outcomes in ATAAD. It reflects circulatory collapse and severe malperfusion, and may assist nonspecialist clinicians in early triage and decision-making.

## Introduction

Surgical outcomes for acute type A aortic dissection (ATAAD) have significantly improved in recent decades, owing to advances in surgical techniques and perioperative care. Despite these gains, certain high-risk subgroups—such as patients presenting with cardiogenic shock due to cardiac tamponade or aortic rupture and those with malperfusion syndromes—continue to experience high mortality.^1–5)^ For these critically ill patients, the initial triage process in the emergency department plays a pivotal role in determining prognosis. Timely decisions, including whether to expedite surgery or perform short-term stabilization, must be made under considerable time pressure, often with limited clinical information.

To support such decisions, several risk stratification tools have been developed to assess the severity of ATAAD and guide treatment strategy.^1–4,6,7)^ Among these, the Penn classification is beneficial as a practical system that accurately reflects the clinical condition, as it classifies patients based on the presence or absence of ischemia and hemodynamic instability.^1–4)^ Nonetheless, applying such classifications early in the clinical course may be challenging, especially for nonsurgical providers who are often the first to assess these patients. Emergency physicians, internists, and cardiology residents may lack the specialized training needed to fully interpret complex imaging or subtle clinical signs in the context of ATAAD.

In this setting, there is a pressing need for simple, objective, and immediately accessible indicators to support early risk evaluation. Blood lactate, a widely used biomarker of tissue hypoperfusion, is one such candidate.^8,9)^ Readily obtainable through point-of-care testing, lactate measurement may provide additional information about the physiological severity of illness in ATAAD patients without requiring advanced clinical interpretation.

The objective of this study is to evaluate whether blood lactate levels at presentation can serve as a practical adjunct to existing classification systems—specifically, the Penn classification—for early risk assessment in ATAAD. By investigating the relationship between initial lactate levels and surgical outcomes, we aim to clarify the potential role of this biomarker in emergency decision-making.

## Materials and Methods

### Materials

The Jichi Medical University Institutional Review Board approved this retrospective observational study and waived the need for informed consent (approval no. 22-172).

**Fig. 1:**
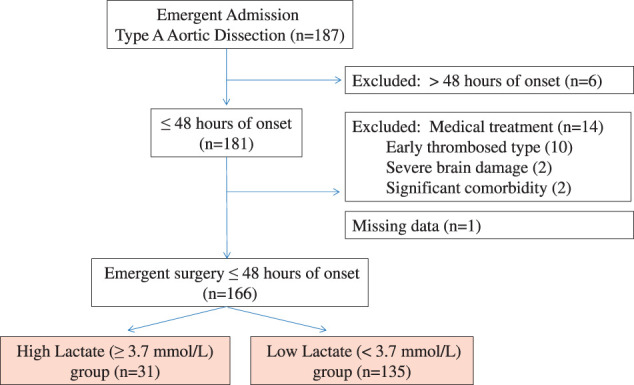
Patient selection process.

Data from 187 consecutive patients with ATAAD who were emergently admitted to the Jichi Medical University Hospital between March 2014 and April 2022 were retrospectively reviewed. The selection process is illustrated in **Fig. 1**. Six patients in whom ATAAD onset occurred after 48 hours were excluded. Of the remaining 181 patients, 14 who received medical treatment and one who had missing lactate level data were excluded. Finally, 166 patients were included (92 men and 74 women; median age and interquartile range, 67 [58–76] years).

### Methods

Preoperative arterial lactate levels were measured on admission to the emergency room. The arterial blood gas analyses were conducted with the GEM Premiere 5000 (Werfen, Barcelona, Spain), and the reference range for arterial lactate concentration is 0.5–2.2 mmol/L.

Thirty-seven preoperative variables, including preoperative arterial lactate levels, were evaluated to determine the risk factors for in-hospital mortality. A univariate analysis was used to evaluate each variable, and variables with p <0.05 were included in the multivariate logistic regression analysis. Regarding the impact of malperfusion, we evaluated patients’ condition in association with the Penn classification to examine the correlation between preoperative arterial blood lactate levels and the Penn class. The Penn classification classified patients with ATAAD into one of the following 4 strata based on clinical presentation: class Aa, absence of ischemia; class Ab, branch vessel malperfusion; class Ac, circulatory collapse; and class Abc, branch vessel malperfusion and circulatory collapse.^1–3)^

Subsequently, we set the cutoff of blood lactate level that affects hospital mortality based on the analysis of the receiver-operating characteristic curve and determined the discriminating lactate levels. Based on the threshold value, we divided the patients into high- and low-lactate groups. The clinical characteristics of the patients in both groups were compared, and the clinical presentation, short- and long-term outcomes, and correlations with Penn class were assessed.

The clinical characteristics of the patients were retrospectively retrieved from their medical records. Mortality was defined as death due to any cause during the follow-up period. Patients were examined at our outpatient clinic or contacted by telephone, and all the patients were followed up. The median follow-up period was 34 months (range, 15–67 months). The follow-up rate was 100%.

### Surgical procedures

Surgeries were performed through median sternotomy with a standard cardiopulmonary bypass. Arterial cannulation sites were the femoral (33%), axillary (13%), ascending (2%), and bidirectional (femoral and axillary arteries) (52%). A cold-blood cardioplegic solution was used for antegrade or retrograde myocardial protection. Open distal anastomosis was performed regularly during circulatory arrest. Antegrade selective or retrograde cerebral perfusion was used for primary cerebral protection during circulatory arrest at a rectal/bladder temperature of 25°C–28°C. The extent of aortic replacement was determined based on the location of the primary entry tear, according to the tear-oriented strategy for patients aged ≥75 years. Aggressive total arch replacement using the frozen elephant trunk (FET) technique was performed in younger patients without hemodynamic instability or multiple organ failure.

### Statistical analyses

Continuous variables are presented as medians (1st quartile; 3rd quartile), and categorical variables are presented as counts. The Mann–Whitney U test was used to compare continuous variables, and the Fisher’s exact test was used to compare categorical variables. The Kruskal–Wallis test with Bonferroni correction was used to compare 3 or more groups. Pairwise comparisons between groups were conducted using the Mann–Whitney U test without adjustment for multiple comparisons. As a result, the potential for increased type I error should be considered when interpreting the results. Multivariate logistic regression analysis was used to identify the preoperative risk factors for in-hospital death. A backward variable selection approach using a p value (p <0.10) was applied. The hazard ratio (HR) with its 95% confidence interval (CI) was estimated for each variable included in the multivariable analysis. The cumulative incidence rates of death were analyzed using the Gray test, accounting for non-aortic deaths, including cancer and senile deaths, as competing risks. Statistical significance was set at p <0.05. All statistical calculations were performed using EZR version 1.53, which is a graphical user interface for R (R Foundation for Statistical Computing, Vienna, Austria).

## Results

### Clinical outcomes

In-hospital mortality occurred in 7 patients (4.2%). The causes of in-hospital mortality included myocardial infarction (2), post-resuscitation encephalopathy (2), mesenteric ischemia (1), cerebral infarction (1), and false lumen rupture of the descending aorta (1). Fifteen patients died after hospital discharge. The causes of remote death included senile death (4), pneumonia (3), malignancy (2), and cerebral infarction (1). The cause was unknown in 5 cases.

### Risk factors for in-hospital death (univariate and multivariate analysis)

Preoperative risk factors for in-hospital mortality are presented in **Table 1**. The univariate analysis revealed that shock (systolic blood pressure <80 mmHg), cardiopulmonary resuscitation, neurological deficits, malperfusion, estimated glomerular filtration rate (eGFR), and preoperative arterial lactate level (mmol/L) were risk factors for in-hospital mortality (p <0.05).

**Table 1:** The preoperative risk factors for in-hospital death (univariate and multivariate analysis)

Variable	Hospital death (n = 7)	Survivors (n = 159)	Univariate p Value	Multivariate		
				HR	95% CI	p Value
Age (years)	66 (54–69)	68 (58–77)	0.415			
Male	6 (86)	86 (54)	0.133			
Comorbidity						
Body mass index	24 (22–29)	24 (22–27)	0.656			
Marfan syndrome	0 (0)	3 (2)	1			
Diabetes	0 (0)	11 (7)	1			
Current smoking	2 (29)	50 (31)	1			
Ischemic heart disease	0 (0)	5 (3.1%)	1			
Cerebrovascular disease	1(14)	13 (8.2)	0.467			
COPD	2 (29)	19 (12)	0.217			
Hemodialysis	0 (0)	3 (1.9)	1			
PAD	0 (0)	1 (0.6)	1			
Redo surgery	0 (0)	1 (0.6)	1			
Preoperative status						
Shock (SBP <80 mmHg)	4 (57)	21 (13)	0.010	2.010	0.237–17.100	0.522
Intubation	1 (14)	9 (5.7)	0.358			
Cardiopulmonary resuscitation	2 (29)	4 (2.5)	0.021	1.920	0.110–33.600	0.6540
Neurological deficit	5 (71)	25 (16)	0.002	7.420	1.220–45.200	0.030
Cardiac tamponade	2 (29)	16 (10)	0.168			
AR ≥moderate	2 (29)	39 (25)	1			
Independence in ADL	7 (100)	155 (98)	1			
DeBakey I	4 (57)	107 (67)	0.686			
DeBakey II	0 (0)	22 (14)	0.596			
DeBakey IIIb retro	3 (43)	30 (19)	0.142			
Early thrombosed	1 (14)	33 (21)	1			
Malperfusion	6 (86)	40 (25)	0.002	19.800	1.410–279.000	0.027
Chemical parameters						
White blood cells	18700 (11650–20400)	11700 (9500–4700)	0.100			
Hemoglobin	12.4 (12.4–13.9)	12.3 (11.3–13.6)	0.419			
Platelets	13.7 (10.6–17.4)	17.0 (13.7–20.3)	0.157			
Albumin	3.7 (3.4–4.0)	3.7 (3.4–4.0)	0.949			
ALT	27 (18–29)	21 (13–36)	0.718			
AST	41 (22–48)	26 (19–40)	0.414			
BUN	20 (19–21)	18 (14–22)	0.326			
eGFR	41 (37–44)	57 (42–71)	0.041	0.964	0.908–1.020	0.227
D-dimer	74 (62–86)	24 (9–53)	0.11			
Total cholesterol	178 (168–193)	171 (148–192)	0.46			
Total protein	6.3 (5.9–6.6)	6.3 (5.9–6.7)	0.69			
LVEF (%)	60 (60–65)	64 (60–70)	0.287			
Lactate (mmol/L)	4.7 (2.8–7.0)	2.1 (1.3–3.1)	0.015	1.400	1.040–1.900	0.029

Data are presented as medians and interquartile ranges or as n (%).

HR: hazard ratio; CI: confidence interval; COPD: chronic obstructive pulmonary disease; PAD: peripheral arterial disease; SBP: systolic blood pressure; AR: aortic regurgitation; ADL: activities of daily living; ALT: alanine aminotransferase; AST: aspartate aminotransferase; BUN: blood urea nitrogen; eGFR: estimated glomerular filtration rate; LVEF: left ventricular ejection fraction

The multivariate risk analysis revealed that neurological deficits (p = 0.030; HR, 7.420; 95% CI, 1.220–45.200), malperfusion (p = 0.027; HR, 19.800; 95% CI, 1.410–279.000), and arterial lactate levels (mmol/L) (p = 0.029; HR, 1.400; 95% CI, 1.040–1.900) were risk factors for in-hospital death. The multivariate risk analysis confirmed that a high preoperative arterial lactate level was a risk factor for in-hospital mortality (**Table 1**).

### Association between the preoperative arterial lactate level and the Penn classification

**Fig. 2:**
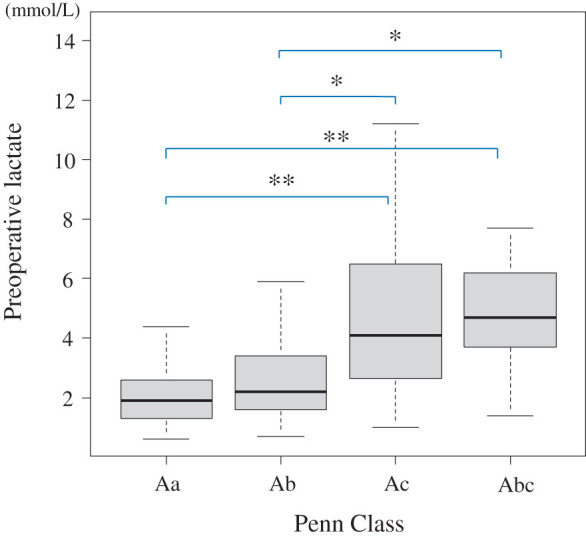
Preoperative lactate levels and the Penn class (Bonferroni correction on Kruskal–Wallis test) Aa vs. Ab, p = 0.220; Aa vs. Ac, p <0.0001; Aa vs. Abc, p = 0.0006; Ab vs. Ac, 0.0199; Ab vs. Abc, 0.0496; Ac vs. Abc, p = 1.0000. *p <0.05; **p <0.001.

The preoperative blood lactate level was 1.9 (1.3, 2.6) mmol/L in Penn class Aa; 2.2 (1.6, 3.4) in class Ab; 4.1 (2.7, 6.5) in class Ac; and 4.7 (3.7, 6.2) in class Abc. The preoperative blood lactate levels in patients with circulatory collapse (Ac) and those with circulatory collapse with malperfusion (Abc) were significantly higher than those in patients without circulatory collapse (Aa and Ab) (**Fig. 2**). The test showed a statistically significant difference among the groups (Kruskal–Wallis chi-squared = 33.356, df = 3, p <0.001).

Thus, the preoperative blood lactate level strongly reflects circulatory collapse with or without malperfusion, indicating serious preoperative conditions.

### A threshold value of serum lactate level that affects hospital mortality

**Fig. 3:**
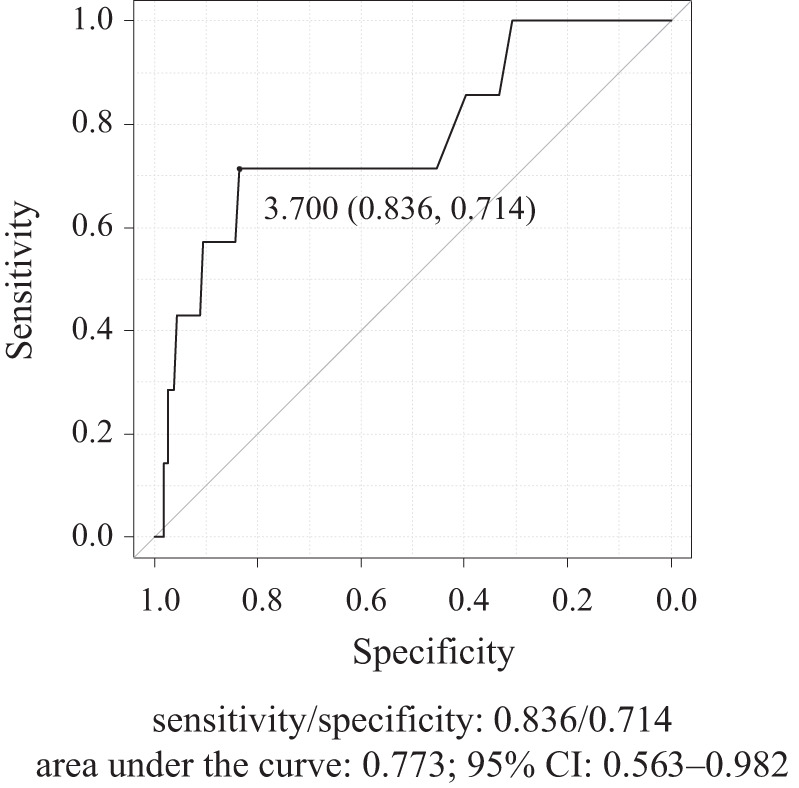
The cut-off blood lactate level, which affects hospital mortality (analysis of the receiver-operating characteristic curve).

We set the cut-off of blood lactate level based on the analysis of the ROC curve to determine the discriminating lactate level, and the threshold was set at 3.7 mmol/L (sensitivity/specificity, 0.836/0.714; area under the curve, 0.773; 95% CI, 0.563–0.982) (**Fig. 3**).

### Patient characteristics and clinical presentations: High-lactate group (≥3.7 mmol/L) vs. low-lactate group (<3.7 mmol/L)

The high-lactate group had a higher body mass index than the low-lactate group. Regarding preoperative status, shock status (systolic blood pressure <80 mmHg), intubation, requirement of cardiopulmonary resuscitation, neurological deficits, cardiac tamponade, coronary malperfusion, renal malperfusion, and multi-organ malperfusion were significantly more frequent in the high-lactate group. The white blood cell count was significantly higher in the high-lactate group than in the low-lactate group. Blood chemistry results indicated that the alanine aminotransferase and aspartate aminotransferase levels and eGFR rate were higher in the high-lactate group than those in the low-lactate group, suggesting the presence of liver and renal dysfunction in the high-lactate group. The left ventricular ejection fraction was significantly lower in the high-lactate group than in the low-lactate group, possibly due to the higher prevalence of coronary malperfusion in the former.

Regarding surgical procedures, there was no significant difference in the extent of proximal or distal aortic replacement between the 2 groups. However, the rates of concomitant coronary artery bypass grafting (CABG) and aortic valve replacement (AVR) were significantly higher in the high-lactate group (**Table 2**).

**Table 2:** Patient characteristics and clinical presentations: high lactate group (≥3.7 mmol/L) vs. low-lactate group (<3.7 mmol/L)

Variable	High-lactate (≥3.7 mmol/L) group (n = 31)	Low-lactate (<3.7 mmol/L) group (n = 135)	p Value
Age (years)	65 (55–77)	68 (58–76)	0.296
Male	22 (71)	70 (52)	0.071
Comorbidity			
Body mass index	24.6 (23.1–29.0)	23.2 (21.7–26.8)	0.031
Marfan syndrome	0	3 (2)	1
Diabetes	2 (6)	9 (6)	1
Current smoking	10 (32)	42 (31)	1
Ischemic heart disease	2 (6)	3 (2)	0.234
Cerebrovascular disease	1 (3)	13 (10)	0.472
COPD	2 (6)	19 (14)	0.372
Hemodialysis	1 (3)	2 (1)	0.464
Peripheral arterial disease	0 (0)	1 (1)	1
Re-do surgery	0 (0)	1 (1)	1
Independence in ADL	31 (100)	131 (97)	1
DeBakey type			
I	18 (58)	93 (69)	0.291
II	5 (16)	17 (13)	0.566
IIIb retrograde	8 (26)	25 (19)	0.453
Early thrombosed	8 (26)	26 (19)	0.338
Preoperative status			
Shock (SBP <80 mmHg)	17 (55)	8 (6)	<0.001
Intubation	6 (19)	4 (3)	0.003
Cardiopulmonary resuscitation	5 (16)	1 (1)	0.001
Neurological deficit	12 (39)	18 (13)	0.003
Cardiac tamponade	13 (42)	5 (4)	<0.001
AR ≥moderate	7 (23)	34 (25)	1
Malperfusion	13 (42)	33 (24)	0.073
Brain	4 (13)	12 (9)	0.503
Coronary	4 (13)	4 (3)	0.041
Mesenteric	2 (6)	1 (1)	0.090
Renal	6 (19)	8 (6)	0.026
Leg	5 (16)	13 (10)	0.336
1-system malperfusion	5 (16)	27 (20)	0.802
≥2-system malperfusion	7 (23)	6 (4)	0.003
Chemical parameter			
White blood cells	15400 (11350–18900)	11200 (8750–13700)	<0.001
Hemoglobin	12.4 (11.3–14.0)	12.3 (11.3–13.6)	0.409
Platelets	15.9 (13.8–19.4)	17.1 (13.6–20.3)	0.507
Albumin	3.8 (2.8–4)	3.7 (3.4–4)	0.600
ALT	28 (20–54.5)	19 (12–32)	0.002
AST	37 (24.5–67.5)	25 (18.5–35)	0.004
BUN	19 (16–23)	18 (13–22)	0.105
eGFR	41.7 (35–57)	57 (45–73)	<0.001
D-dimmer	35 (11–56)	23 (10–51)	0.715
Total cholesterol	169 (143–184)	172 (149–193)	0.613
Total protein	6.2 (5.8–6.6)	6.4 (5.9–6.7)	0.246
LVEF (%)	60 (60–65)	65 (60–70)	0.002
Intraoperative variables			
Operation time (min.)	423 (379–477)	405 (354–467)	0.371
Cardiopulmonary bypass time (min)	220 (181–256)	213 (176–249)	0.360
Surgery			
Ascending aorta replacement	24 (77)	102 (76)	1
+ Arch replacement	7 (23)	33 (24)	1
+ Root replacement	5 (16)	11 (8)	0.184
+ AVR	4	7	0.020
+ CABG	5	6	0.033

Data are presented as medians and interquartile ranges or as n (%).

COPD: chronic obstructive pulmonary disease; ADL: activities of daily living; SBP: systolic blood pressure; AR: aortic regurgitation; ALT: alanine aminotransferase; AST: aspartate aminotransferase; BUN: blood urea nitrogen; eGFR: estimated glomerular filtration rate; LVEF: left ventricular ejection fraction; AVR: aortic valve replacement; CABG: coronary artery bypass grafting

### Short-term outcomes and relation with the Penn class

Hospital mortality was significantly higher in the high-lactate group than in the low-lactate group (16% vs. 1%, p = 0.003), and the intensive care unit (ICU) stay was significantly longer in the high-lactate group than in the low-lactate group [12 (8–22) vs. 7 (5–9) days, p <0.001) Major complications, including neurological deficits, pneumonia, and the requirement of postoperative extracorporeal membrane oxygenation and blood purification, were more frequent in the high-lactate group (**Table 3**).

**Table 3:** Short-term surgical outcomes: high-lactate group (≥3.7 mmol/L) vs. low-lactate group (<3.7 mmol/L)

Variable	High-lactate (≥3.7 mmol/L) group (n = 31)	Low-lactate (<3.7 mmol/L) group (n = 135)	p Value
Hospital death	5 (16)	2 (1)	0.003
Hospital stay (days)	32 (16–52)	22 (16–31)	0.074
ICU stay (days)	12 (8–22)	7 (5–9)	<0.001
Major complications			
Neurological deficit	7 (23)	7 (5)	0.005
Pneumonia	7 (23)	6 (4)	0.003
Tracheostomy	4 (13)	5 (4)	0.064
Mesenteric ischemia	2 (6)	1 (1)	0.090
Paraplegia	1 (3)	2 (1)	0.464
Mediastinitis	0 (0)	2 (1)	1
Re-exploration	1 (3)	4 (3)	0.566
Postoperative ECMO	6 (19)	1 (1)	<0.001
Postoperative blood purification	8 (26)	14 (10)	0.036
Penn classification			
Class Aa	8 (26)	97 (72)	<0.001
Class Ab	6 (19)	30 (22)	0.813
Class Ac	10 (32)	6 (4)	<0.001
Class Abc	7 (23)	2 (1)	<0.001

Data are presented as medians and interquartile ranges or as n (%).

ICU: intensive care unit; ECMO: extracorporeal membrane oxygenation

Regarding the Penn class, classes Ac and Abc were more frequent in the high-lactate group than in the low-lactate group (p <0.001). In contrast, class Aa (absence of branch vessel malperfusion or circulatory collapse) was rare in the high-lactate group (p <0.001). The rate of malperfusion without circulatory collapse (Ab) did not differ between the groups. Therefore, a threshold value of 3.7 mmol/L identified high-risk patients.

### Long-term outcomes

**Fig. 4:**
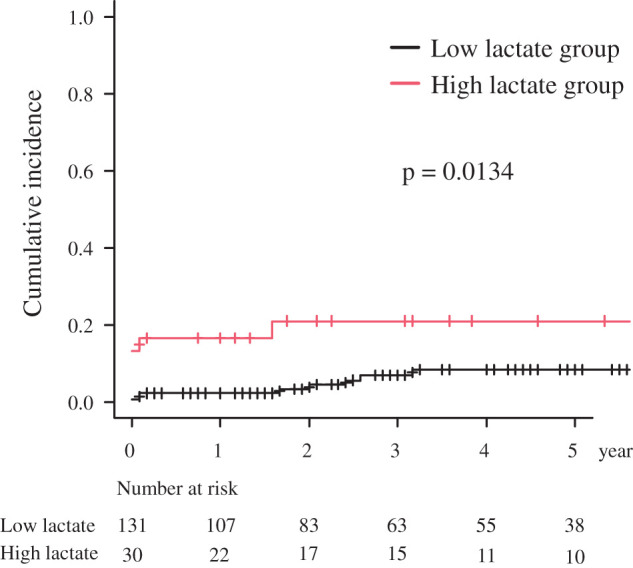
The cumulative incidence rate of death (accounting for death from malignancy or senility as a competing risk).

The cumulative incidence rate of death (accounting for death from malignancy or senility as a competing risk) was significantly higher in the high-lactate group (0.211 at 5 years) than that in the low-lactate group (0.120) (p = 0.013) (**Fig. 4**).

## Discussion

This study demonstrates that elevated preoperative arterial lactate levels, particularly those ≥3.7 mmol/L, are significantly associated with increased in-hospital mortality and adverse long-term outcomes following emergency surgery for ATAAD. Our findings highlight the utility of lactate as a rapid, objective, and easily accessible biomarker that reflects the severity of circulatory compromise and malperfusion, particularly in patients classified as Penn Ac and Abc.

Risk stratification tools such as EuroSCORE II^6)^ and the GERAADA (German Registry of Acute Aortic Dissection Type A) risk score^7)^ have been proposed to estimate postoperative mortality in ATAAD patients. Although these models offer good predictive accuracy, they rely on detailed imaging and clinical evaluations, which may delay urgent surgical decision-making. Similarly, the Penn classification,^1–3)^ one of the most validated systems for preoperative prognostication, requires clinical expertise and diagnostic interpretation that may be beyond the capabilities of nonspecialists or residents at the time of initial presentation.

**Fig. 5:**
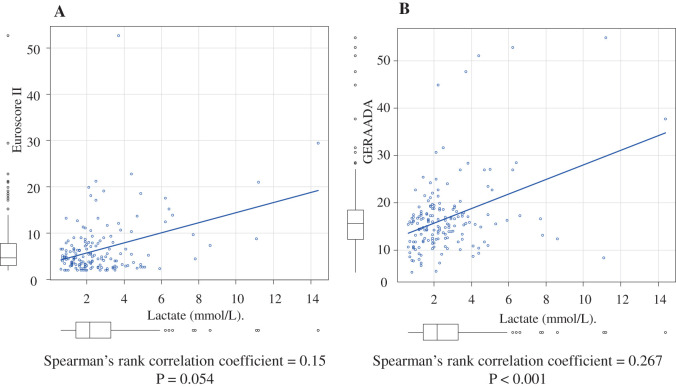
(**A**) Spearman’s rank correlation between preoperative lactate level and EuroSCORE II. (**B**) Spearman’s rank correlation between preoperative lactate level and GERAADA score. GERAADA: German Registry of Acute Aortic Dissection Type A

In our study, preoperative lactate levels showed a significant correlation with the GERAADA score (Spearman’s ρ = 0.267, p = 0.000518), while the correlation with EuroSCORE II was weaker and did not reach statistical significance (Spearman’s ρ = 0.15, p = 0.0541) (**Fig. 5**). This difference likely reflects the design focus of each model: GERAADA is specific to ATAAD and incorporates indicators of malperfusion and hemodynamic instability—factors closely associated with elevated lactate. Furthermore, lactate levels also correlated well with the Penn classification, reinforcing the view that lactate serves not only as a marker of systemic hypoperfusion but also as a surrogate for disease severity in ATAAD. These findings support the value of lactate as a simple, quantifiable parameter that aligns with more complex, established risk models.

Blood lactate concentration offers a simple point-of-care alternative, readily available in any emergency setting.^8,9)^ It directly reflects systemic hypoperfusion and, as shown in our analysis, correlates strongly with Penn classes associated with high mortality risk. Notably, we identified a threshold of 3.7 mmol/L as the optimal cut-off for predicting poor outcomes. Patients exceeding this threshold exhibited not only higher in-hospital mortality but also a significantly greater incidence of perioperative complications, prolonged ICU stays, and long-term mortality.

Previous studies have reported associations between preoperative hyperlactatemia and mortality in ATAAD.^10–15)^ However, these studies varied in their proposed threshold values and in the strength of correlation with clinical classifications such as the Penn system. For example, Gemelli et al.^11)^ and Zindovic et al.^13)^ did not find a strong correlation between lactate and Penn class, possibly due to lower thresholds (2.85 and 2.2 mmol/L, respectively) that may not have adequately identified patients with profound circulatory collapse or mesenteric ischemia. In contrast, our findings suggest that a higher cutoff value more accurately reflects critical physiological deterioration and better aligns with the most severe Penn categories (Ac and Abc).

Recent efforts to improve prognostic accuracy have included composite biochemical risk models that integrate lactate with other parameters such as creatinine or liver enzymes.^14,15)^ While such models may offer incremental predictive value, they are often impractical in emergent situations due to delays in obtaining full laboratory data. Our results indicate that lactate alone, which can be measured within minutes, provides sufficient discriminatory power for emergency triage and early clinical decision-making.

We also examined the relationship between preoperative lactate levels and the surgical procedures performed. Although there was no significant difference in the extent of proximal or distal aortic replacement between the groups, concomitant CABG and AVR were more frequently performed in patients with high lactate levels. This likely reflects the presence of coronary malperfusion or aortic regurgitation contributing to peripheral circulatory failure, and suggests that elevated lactate may influence intraoperative strategy. Importantly, this study includes cases treated before the widespread adoption of the FET procedure. In current practice, FET is frequently used in patients with true lumen collapse and associated organ ischemia—clinical scenarios that are typically accompanied by elevated lactate levels. Thus, preoperative lactate may serve not only as a prognostic marker but also as a useful guide when selecting surgical strategies, including the use of FET.

Although the long-term outcomes between the 2 groups appeared similar, the difference in early mortality indicates a significant clinical impact of the initial perioperative period. This finding suggests that optimal surgical management and perioperative care are critical in improving early survival. Furthermore, once patients overcome the initial high-risk phase, their long-term prognosis tends to be favorable. Therefore, presenting these outcomes provides a comprehensive understanding of the overall impact of surgical treatment.

### Limitations

This study is not without limitations. It was a retrospective, single-center analysis with a relatively small cohort, which may limit the generalizability of our findings. Additionally, we used a single lactate measurement at admission, precluding analysis of lactate kinetics or clearance, which might offer additional prognostic insight. Finally, lactate levels can be influenced by various patient-specific factors and comorbidities. Despite these limitations, the consistent association between elevated lactate and both short- and long-term adverse outcomes supports its role as a practical risk stratification tool in ATAAD.

### Clinical implications

The findings of this study have important clinical implications. Preoperative lactate levels can help identify high-risk patients early in their clinical course, enabling timely surgical planning, targeted resuscitation strategies, or consideration of adjunctive therapies. Importantly, this biomarker can be effectively utilized by nonspecialists in the emergency setting, offering a bridge between initial triage and definitive surgical intervention.

## Conclusion

Preoperative lactate levels ≥3.7 mmol/L served as a strong surrogate marker for predicting surgical outcomes in patients with ATAAD. These elevated levels corresponded closely with Penn classes Ac and Abc—indicators of circulatory collapse with or without malperfusion—reflecting critical preoperative status. As a simple and rapidly obtainable biochemical marker, lactate is well suited for use in emergency settings and may aid in both early risk stratification and the formulation of appropriate surgical strategies.

## Declarations

### Data Availability

The data that support the findings of this study are available from the corresponding author upon reasonable request.
